# Development and Field Evaluation of a Sustainable Polymer–Mineral Modified Bitumen Binder for Asphalt Pavements

**DOI:** 10.3390/ma19173768

**Published:** 2026-09-04

**Authors:** Aidar Kengesbekov, Alfira Sabitova, Temirlan Kusainov, Zhanna Sharipkhan, Gulzat Aitkaliyeva, Moldir Ualkhanova

**Affiliations:** 1 Institute of Composite Materials, Ust-Kamenogorsk 070000, Kazakhstan; kenesbekovaidar@gmail.com (A.K.); kusainov.temirlan00@mail.ru (T.K.); 2 Department of Chemistry and Ecology, Research School of Physical and Chemical Sciences, Shakarim University NJSC, Semey 071412, Kazakhstan; alfa-1983@mail.ru (A.S.); zhanka_81@mail.ru (Z.S.); 3 Department of Chemical and Biochemical Engineering, Geology and Oil-Gas Business Institute Named After K. Turyssov, Satbayev University NJSC, Almaty 050013, Kazakhstan; g.aitkaliyeva@satbayev.university

**Keywords:** modified bitumen, SBS polymer, oil sludge, titanium slag, cyclone dust, metallurgical by-products, asphalt concrete, FTIR, TGA/DSC, XRD

## Abstract

The development of more sustainable asphalt materials requires modified bitumen binders that combine thermal resistance, adhesion, and deformation capacity. In this study, soft paving-grade bitumen was modified with styrene–butadiene–styrene polymer, oil refinery sludge, titanium slag, and lead-furnace cyclone dust. Binders with bitumen-to-modifying-system ratios from 90:10 to 60:40 were characterized by FTIR, TGA/DSC, XRD, and portable XRF analyses and evaluated using penetration-type consistency, softening-point, qualitative adhesion, and tensile deformation tests. The modifying-system content produced non-monotonic changes in binder properties. Among the investigated formulations, the 75:25 composition provided the most balanced response, with a penetration depth of 8.6 mm, a softening point of 65.0 °C, a maximum elongation of 355.66 mm, and high qualitative film retention on mineral aggregate. In contrast, the 60:40 binder showed increased penetration and the lowest elongation, indicating that excessive modifier loading was unfavorable. The 75:25 binder was subsequently used in small-scale asphalt concrete sections and showed acceptable preliminary behavior during mixture preparation, placement, and compaction. The results demonstrate composition-dependent relationships in the investigated multicomponent system and support further evaluation of selected industrial by-products in modified bitumen binders.

## 1. Introduction

Asphalt pavements are exposed to traffic loading, temperature variations, moisture, and oxidative aging. These factors contribute to rutting, cracking, stripping, raveling, and other forms of premature deterioration. Since asphalt concrete is a bitumen–mineral composite, binder properties strongly influence resistance to high-temperature deformation, low-temperature cracking, fatigue damage, moisture-induced deterioration, and aging [1,2,3].

Bitumen is a complex organic material composed mainly of saturates, aromatics, resins, and asphaltenes. The relative proportions and associations of these fractions determine its colloidal organization, consistency, viscosity, temperature sensitivity, and deformation behavior [4,5]. The maltene phase contributes to mobility and deformability, whereas asphaltene-rich domains provide structural rigidity. Disturbance of this balance may result in either excessive softening or excessive stiffening and embrittlement. Aging further changes the binder through volatilization of lighter fractions, formation of oxygen-containing groups, and increased association of polar components, generally increasing stiffness while reducing ductility and relaxation capacity [3,4,5]. Effective modification should therefore improve resistance to thermal and mechanical loading without causing an excessive loss of deformability.

This balance is particularly important for soft paving-grade binders such as MGO 130/200. Although such binders provide favorable workability and deformation capacity, they may show insufficient resistance to flow and permanent deformation at elevated temperatures. The modification approach adopted in the present study was based on the combined introduction of an elastomeric polymer, an organic refinery by-product, and metallurgical mineral constituents.

Styrene–butadiene–styrene (SBS) block copolymer is widely used for bitumen modification because it absorbs compatible bitumen fractions, swells, and may form a polymer-rich network. Polystyrene domains act as physical junctions, whereas polybutadiene segments provide flexibility and elastic response, thereby improving recovery behavior and resistance to permanent deformation [4,5,6]. However, SBS modification may increase viscosity and processing temperature and may lead to phase separation or storage instability. Its effectiveness depends on the composition of the base bitumen and on the presence of additional organic and mineral constituents.

Oil refinery sludge is an industrial by-product that may contain hydrocarbons, water, oxidized compounds, mineral matter, and mechanical impurities [7,8]. Its organic fraction may interact with the maltene phase and influence binder consistency, plasticity, and colloidal balance. At moderate contents, it may partly compensate for polymer- or filler-induced stiffening. However, its composition varies with source and storage conditions, and excessive addition may produce softening, heterogeneity, or reduced compatibility. Therefore, refinery sludge cannot be regarded as a universal rejuvenating agent and must be evaluated within each specific binder system.

Titanium slag and lead-furnace cyclone dust were used as metallurgical components of the modifying system. These materials may act as dispersed fillers, increase the inorganic contribution, and affect consistency, interfacial interaction, thermal response, and stress distribution [9,10]. At the same time, excessive mineral loading may promote particle agglomeration, increase heterogeneity, interfere with polymer swelling, and reduce deformation capacity. Because cyclone dust contains metal-bearing constituents, its potential application also requires elemental characterization and subsequent environmental assessment, particularly with regard to heavy-metal mobility and leaching.

Previous studies have generally investigated SBS polymers, petroleum-derived organic additives, and mineral industrial by-products as separate bitumen-modification approaches [5,6,7,8,9,10]. Less attention has been given to binders containing all three types of components simultaneously. In such multicomponent systems, the effects may be complementary, neutral, or adverse. SBS may provide polymer reinforcement, oil sludge may regulate the organic phase, and mineral particles may contribute to dispersed structuring. Conversely, unsuitable proportions may restrict polymer swelling, increase compositional heterogeneity, or create an unfavorable balance between consistency and deformability.

From a mechanistic standpoint, the components of the modifying system may affect different levels of binder organization. SBS can absorb compatible maltene fractions and swell within the bitumen matrix [4,5,6], whereas the hydrocarbon-containing fraction of oil sludge may influence the solvating environment of the maltene phase and thereby affect polymer swelling and colloidal balance [7,8]. The introduced mineral particles may contribute through surface and interfacial interactions, while excessive mineral loading may interfere with polymer swelling and increase compositional heterogeneity [9,10]. Because wettability, surface energy, phase morphology, and specific intermolecular interactions were not measured directly in the present study, hydrophobic–hydrophilic effects and component compatibility cannot be quantified independently. Accordingly, this mechanism is considered a physically based interpretation of the multicomponent system rather than direct evidence of specific molecular interactions.

The scientific question addressed in this study was how the total content of a fixed SBS–oil sludge–mineral modifying system affects the physicochemical characteristics and conventional properties of soft paving-grade bitumen. It was hypothesized that an intermediate modification level would provide a more favorable balance by retaining a substantial organic binder contribution while allowing polymer reinforcement and dispersed mineral structuring. Insufficient modification was expected to provide limited changes, whereas excessive modifier loading was expected to impair deformation performance and increase heterogeneity.

The experimental design was intended to establish composition-dependent relationships within the investigated multicomponent system. Separate single-modifier and binary reference binders were not included; therefore, the study does not claim to demonstrate true synergy or quantify the independent contribution of each component. Instead, it evaluates the combined influence of the complete modifying system. FTIR spectroscopy, TGA/DSC, XRD, and portable XRF analyses were interpreted together with penetration-type consistency, softening-point, tensile deformation, and qualitative adhesion results.

Accordingly, the aim of this study was to determine how modifying-system content influences the physicochemical characteristics, thermal behavior, consistency, adhesion, and tensile deformation capacity of a polymer–organic–mineral binder based on MGO 130/200 bitumen, SBS, refinery sludge, titanium slag, and lead-furnace cyclone dust. Binders with different bitumen-to-modifying-system ratios were compared to identify the most balanced formulation among those investigated. The selected binder was subsequently used in small-scale asphalt concrete sections for a preliminary assessment of its technological applicability during mixture preparation, placement, and compaction.

## 2. Materials and Methods

### 2.1. Materials

Soft paving-grade bitumen MGO 130/200 manufactured by BITUM LLC (Salavat, Republic of Bashkortostan, Russia) was used as the base binder, and styrene–butadiene–styrene block copolymer (SBS) manufactured by SIBUR (Moscow, Russia) was used as the polymer modifier. Oil refinery sludge obtained from the Pavlodar Petrochemical Plant (Pavlodar, Kazakhstan) was employed as an organic-rich industrial by-product. Titanium slag obtained from the Ust-Kamenogorsk Titanium and Magnesium Plant (Ust-Kamenogorsk, Kazakhstan) and lead-furnace cyclone dust obtained from Kazzinc (Ust-Kamenogorsk, Kazakhstan) were used as metallurgical by-products containing mineral constituents.

Before incorporation into the binder, the oil refinery sludge, titanium slag, and lead-furnace cyclone dust were dried to reduce residual moisture. Coarse agglomerates, where present, were mechanically disintegrated, and the materials were sieved to remove oversized particles and foreign inclusions. This preliminary treatment was intended to reduce moisture and particle-size heterogeneity and to facilitate the dispersion of the components during subsequent hot blending with bitumen.

Detailed standalone XRD and XRF characterization of the individual industrial by-products was not performed within the original experimental program. Therefore, their complete phase and bulk elemental compositions are not reported in the present study. The available characterization of these materials is limited to the analytical data presented in the following sections.

### 2.2. Preparation of Modified Bitumen Binders

**Figure 1:**
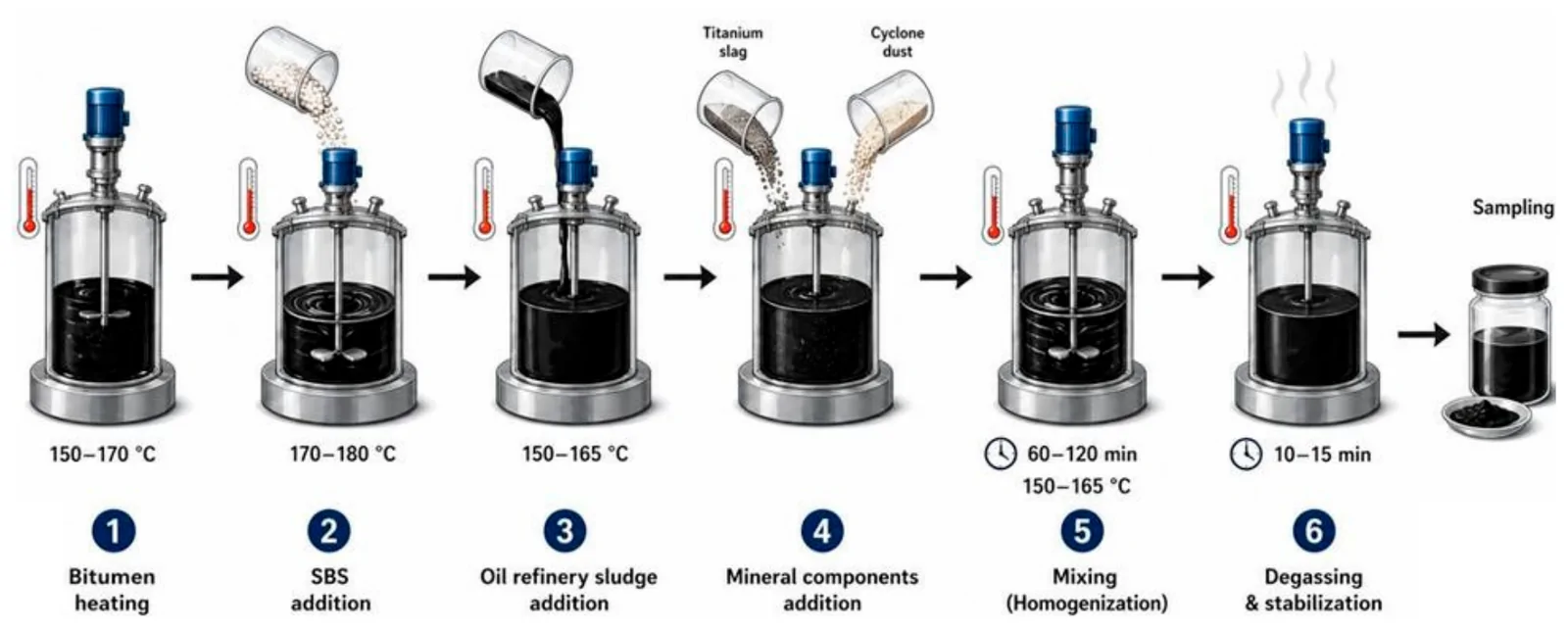
Technological scheme for preparing polymer–organic–mineral modified bitumen binders.

The polymer–organic–mineral modified bitumen binders were prepared by sequentially introducing the modifying components into the heated base bitumen, as illustrated in Figure 1. The base bitumen was heated to 150–170 °C under continuous stirring until it became sufficiently fluid for the incorporation of the modifiers.

The temperature was then maintained within the range of 170–180 °C, and SBS was gradually introduced under continuous stirring. The polymer was added in small portions to reduce the formation of visible agglomerates and to facilitate its swelling and dispersion within the bitumen.

After SBS incorporation, the mixture temperature was adjusted to 150–165 °C, and the oil refinery sludge was gradually added under continuous stirring. Titanium slag and lead-furnace cyclone dust were subsequently introduced stepwise. The metallurgical by-products were added in small portions to reduce local particle accumulation during mixing.

The total preparation time ranged from 60 to 120 min, depending on the formulation. After completion of mixing, the prepared binders were maintained at the processing temperature for 10–15 min to allow entrained air bubbles to escape. The binders were then poured into appropriate molds or containers, cooled, and stored for subsequent physicochemical and physical–mechanical testing.

### 2.3. Composition of Modified Bitumen Binders

The investigated binders were prepared by varying the mass ratio between the base bitumen and the total modifying system. The control sample consisted of unmodified MGO 130/200 bitumen. The modified binders were designated as 90:10, 80:20, 75:25, 70:30, 65:35, and 60:40, where the first number represents the mass fraction of the base bitumen and the second number represents the total mass fraction of the modifying system.

The total modifying system consisted of SBS, oil refinery sludge, titanium slag, and lead-furnace cyclone dust. Its content was varied from 10 to 40 wt.% to evaluate the composition-dependent response of the multicomponent binders. The detailed compositions are presented in Table 1.

The selected formulations covered low, intermediate, and high total modifying-system contents. This compositional range was used to assess whether changes in modifier loading produced a monotonic or non-monotonic response in the physicochemical and conventional binder properties. The 60:40 composition represented the highest modifier loading investigated in the present study.

### 2.4. FTIR Analysis

Fourier-transform infrared spectroscopy was used for the qualitative identification of the principal functional groups in the raw components and for comparison of the control bitumen with the selected 75:25 modified binder [11,12,13]. The measurements were performed using an FT-801 FTIR spectrometer (Simex, Novosibirsk, Russia).

The samples were prepared as potassium bromide pellets using a sample-to-KBr mass ratio of 1:10. Before pellet preparation, the potassium bromide was ground and dried at 200 ± 5 °C for 3 h to remove adsorbed moisture.

The spectra were recorded over the wavenumber range of 500–4000 cm^−1^ at a spectral resolution of 1 cm^−1^. Each spectrum was obtained from 100 scans at 25 ± 2 °C.

The FTIR spectra were used to identify characteristic absorption bands associated with aliphatic C–H bonds, aromatic structures, oxygen-containing functional groups, and inorganic metal–oxygen vibrations. The spectrum of the 75:25 binder was compared with that of the control bitumen to evaluate changes in the principal hydrocarbon absorption bands and possible oxidation-related spectral features. The FTIR results were interpreted qualitatively and were not used for quantitative determination of component contents, phase distribution, or chemical conversion.

### 2.5. XRD Analysis

X-ray diffraction was used for the qualitative comparison of the structural features of the control bitumen and the polymer–organic–mineral modified binders. The measurements were performed using an X’Pert PRO diffractometer (PANalytical, B.V., Almelo, The Netherlands) with monochromated Cu Kα radiation. The wavelength of the Cu Kα_1_ radiation was 1.54187 Å.

The samples were analyzed in reflection geometry using a rectangular aluminum multipurpose sample holder (PW1172/01, PANalytical B.V., Almelo, The Netherlands). The diffraction patterns were recorded over the 2θ range of 10–40°. The X-ray tube voltage and current were 45 kV and 30 mA, respectively. A step size of 0.05° and a counting time of 10 s per step were used.

The diffraction patterns were evaluated qualitatively to compare the broad amorphous contribution of the organic binder phase with the crystalline reflections associated with the introduced metallurgical constituents. No quantitative phase analysis was performed, and the XRD data were not used to determine particle distribution or phase continuity within the binders.

### 2.6. TGA/DSC Analysis

Simultaneous thermogravimetric and differential scanning calorimetry analysis was used to evaluate the thermal behavior of the investigated binders. The measurements were performed using a Labsys Evo thermal analyzer (Setaram, Caluire-et-Cuire, France) under an argon atmosphere.

The samples were heated from approximately 30 to 1000 °C at a heating rate of 10 ± 1 °C min^−1^. The initial sample mass was approximately 25 ± 2 mg.

The TGA curves were used to compare the principal mass-loss regions and the residual mass remaining at the end of the heating program. The accompanying DSC curves were used for qualitative comparison of thermal events occurring during heating. Because individual thermal events may overlap in the multicomponent binders, the DSC features were not assigned unambiguously to specific reactions or individual components.

The thermal data were interpreted qualitatively to compare the control bitumen and selected modified binders. The residual mass was considered to represent the combined contribution of thermally stable inorganic constituents and possible carbonaceous products remaining under the applied inert-atmosphere conditions.

### 2.7. XRF Analysis

X-ray fluorescence analysis was used for the semi-quantitative comparison of the surface elemental profiles of the modified bitumen binders. The measurements were performed using a handheld Hitachi X-MET8000 Smart XRF analyzer (Hitachi High-Tech Analytical Science, Tokyo, Japan).

Before analysis, each sample was placed on a flat surface, and the measurement area was selected so that the analyzer window was in full contact with the sample surface. The analyzer was held stationary throughout the acquisition. The measurement time was 10 s per analyzed point.

The analysis focused on elements associated with the introduced metallurgical by-products, including Pb, Ti, Fe, Cd, Zn, As, Cr, Zr, V, Bi, and Cu. The instrument-reported relative elemental values were used to compare the Pb-, Ti-, Fe-, Zn-, and other metal-containing contributions among the modified binders.

Because a handheld XRF analyzer primarily characterizes the analyzed surface region, the results were interpreted as semi-quantitative relative values rather than bulk elemental concentrations. The method was not used to determine the chemical state, spatial distribution, environmental mobility, or leachability of the detected elements.

### 2.8. Penetration-Type Consistency Test

The consistency of the control and modified bitumen binders was evaluated using a needle penetration-type test based on the general principle of GOST 11501-78 [14]. The measurements were performed using a TA-3000 texture analyzer (Saicheng, Jinan, China) equipped with a needle probe.

Before testing, the binder samples were conditioned at 25 ± 2 °C until temperature stabilization. During the measurement, the probe was moved vertically into the sample under instrument-controlled conditions, while the penetration depth and material resistance were recorded.

The penetration depth was reported in millimetres as directly registered by the texture analyzer. Therefore, the reported values represent penetration depths obtained using the adapted instrumental procedure rather than the conventional penetration index expressed in units of 0.1 mm according to GOST 11501-78.

The results were used for comparative evaluation of binder consistency under the applied test conditions. Lower penetration depths were interpreted as greater resistance to local needle penetration, whereas higher values indicated a softer local consistency. The test was not used as a direct measurement of rheological stiffness or complete mechanical performance.

### 2.9. Softening Point

The softening point of the control and modified bitumen binders was determined using the ring-and-ball method in accordance with GOST 11506-73 [15].

Before testing, the binder was heated at 160 °C for 50 min until it reached a sufficiently fluid state and was then poured into metal rings with a slight excess. After cooling, the excess binder was removed with a spatula to obtain a flat sample surface. The prepared rings were fixed in the holder, immersed in the test bath, and steel balls were placed on the binder surfaces.

The bath temperature was increased at an average heating rate of approximately 7 °C min^−1^. The softening point was recorded when the steel ball and the softened binder reached the lower reference level. Two parallel measurements were performed for each composition, and the mean value was reported.

The obtained values were used to compare the susceptibility of the binders to softening under the applied ring-and-ball test conditions. The softening point was not interpreted as an independent measure of complete rheological or pavement performance.

### 2.10. Tensile Deformation Test

The tensile deformation behavior of the control and modified bitumen binders was evaluated using an adapted procedure based on the general principle of the bitumen ductility test described in GOST 11505-75 [16]. The measurements were performed using an AGS-X 100 kN universal testing machine (Shimadzu Corporation, Kyoto, Japan) operated in tensile mode.

The binder specimens were molded and conditioned at 25 ± 2 °C until temperature stabilization. Each specimen was then fixed in the grips of the testing machine and subjected to tensile deformation under controlled displacement. During testing, the grip displacement, test duration, and moment of specimen failure were recorded.

The maximum grip displacement registered before specimen failure was reported in millimetres and used as a comparative indicator of tensile deformation capacity. Higher values indicated a greater ability of the specimen to sustain extension under the applied test conditions.

Because the measurements were performed using an adapted universal-testing-machine procedure, the reported values should not be regarded as conventional ductility values obtained using a standard bitumen ductilometer. The results were used only for comparative evaluation of the control and modified binders and were not interpreted as a complete measure of low-temperature cracking or fatigue resistance.

### 2.11. Qualitative Adhesion Assessment

Adhesion was evaluated qualitatively using crushed-stone aggregate. The test was performed to assess the ability of the modified binder film to remain attached to the mineral surface after water–thermal exposure. After exposure, the specimens were visually examined for retention, local damage, and peeling of the bitumen film.

The results were used for comparative evaluation of binder-film retention on the aggregate surface under the applied test conditions. This qualitative assessment was not interpreted as a direct measurement of the moisture susceptibility or stripping resistance of the asphalt concrete mixture.

### 2.12. Preparation and Preliminary Evaluation of Small-Scale Asphalt Concrete Sections

After the laboratory comparison of the investigated binders, the 75:25 composition was selected as the most balanced formulation under the adopted evaluation criteria and was used to prepare a small-scale experimental asphalt concrete section.

Two comparative sections with approximate dimensions of 50 × 50 cm were prepared: a control section containing the base MGO 130/200 bitumen and an experimental section containing the selected 75:25 modified binder. Both sections were constructed using the same general preparation, placement, and compaction procedure.

The supporting base consisted of compacted crushed stone–sand material. A coarse-grained asphalt concrete layer with a thickness of approximately 6–7 cm was placed over the prepared base. After placement and compaction, the sections were visually inspected for obvious surface defects.

Surface macrotexture was assessed using an adapted sand patch procedure based on ST RK 1279-2013 [17]. A fixed volume of 25 cm^3^ of dry sand was distributed over the cleaned surface, and the diameter of the resulting patch was measured in four intersecting directions. One sand patch was evaluated on each section. The mean patch diameter and mean texture depth were calculated as described in Section 3.8.

**Figure 2:**
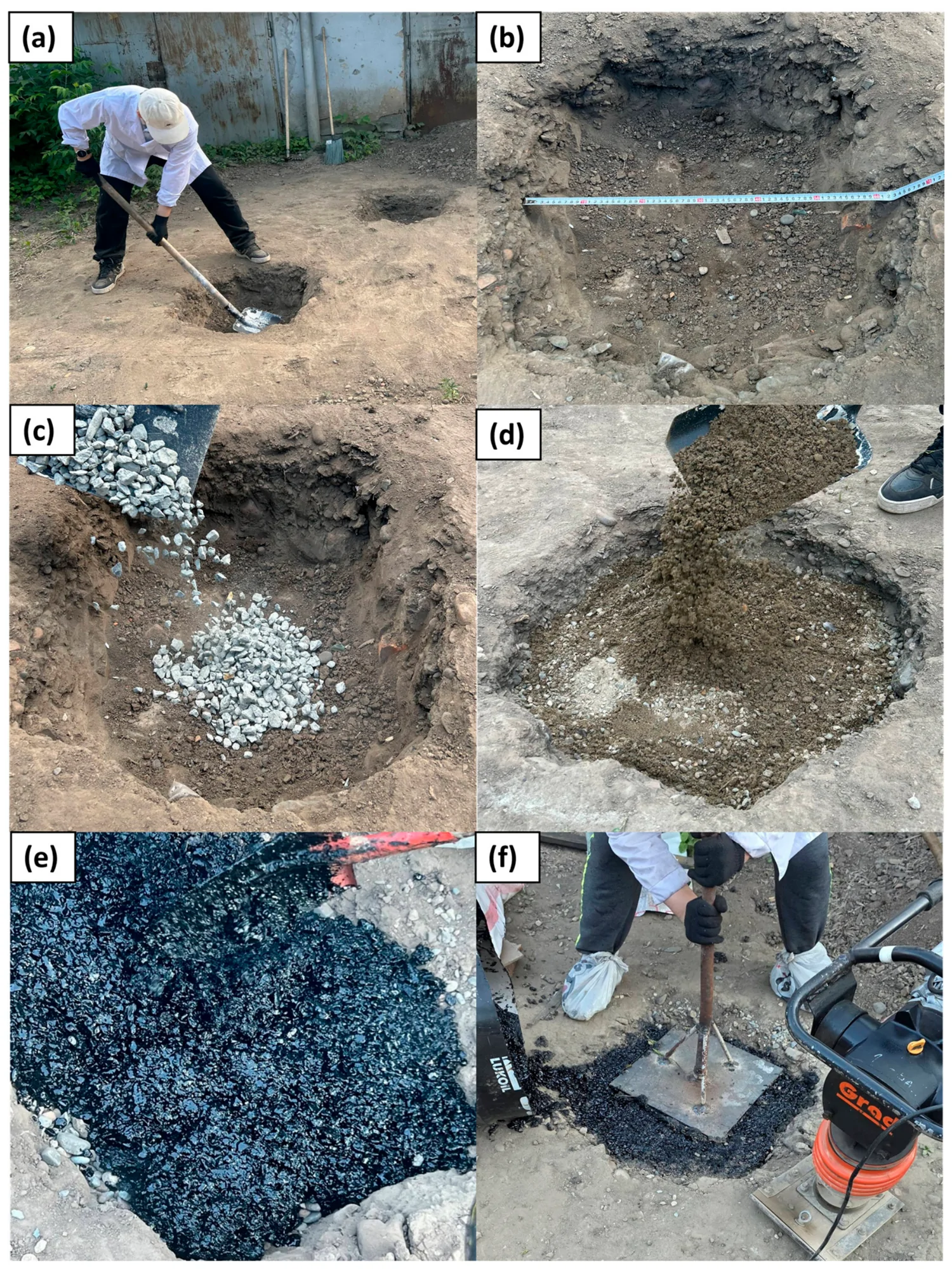
Preparation and field evaluation of experimental asphalt concrete sections: (**a**) base preparation; (**b**) dimensional control; (**c**) crushed-stone layer placement; (**d**) sand–gravel base placement; (**e**) asphalt concrete mixture placement; (**f**) compaction.

The preparation and field evaluation of the experimental asphalt concrete sections are shown in Figure 2.

### 2.13. Use of Generative Artificial Intelligence

The schematic illustration presented in Figure 1 was generated using ChatGPT Images 2.0 (OpenAI, San Francisco, CA, USA; accessed in June 2026). The tool was used solely to create a visual representation of the technological sequence for preparing the polymer–organic–mineral modified bitumen binder. The experimental procedure, processing stages, sequence of component addition, material designations, temperature ranges, and processing durations shown in the figure were defined by the authors based on the actual experimental work.

The generated output was reviewed and verified by the authors for consistency with the experimental procedure. The artificial intelligence tool was not used to generate, modify, analyze, or interpret experimental data, numerical results, analytical graphs, tables, or scientific conclusions. The authors take full responsibility for the accuracy and content of Figure 1.

## 3. Results and Discussion

### 3.1. FTIR Analysis

**Figure 3:**
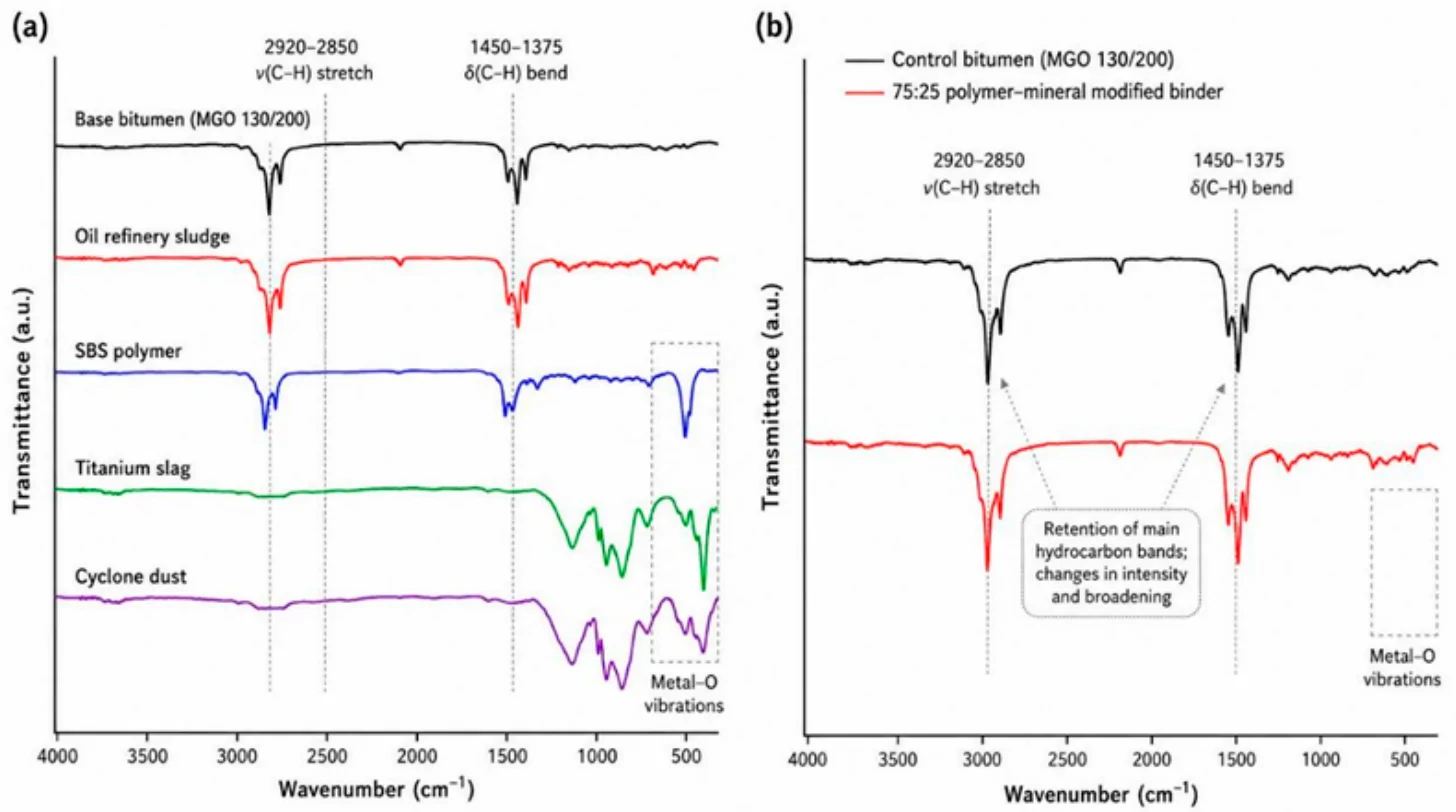
FTIR spectra of: (**a**) the raw components, including base bitumen MGO 130/200, oil refinery sludge, SBS polymer, titanium slag, and lead-furnace cyclone dust; and (**b**) the control bitumen and the selected 75:25 modified binder.

The FTIR spectra of the raw components, control bitumen, and the selected 75:25 polymer–organic–mineral binder are shown in Figure 3. The spectra of the raw materials were examined to identify their principal absorption regions, while the comparison between the control bitumen and the 75:25 binder was used to evaluate qualitative changes in the chemical environment and spectral characteristics after modification.

As shown in Figure 3a, the base bitumen exhibits absorption bands characteristic of hydrocarbon binders. The intense bands in the approximately 2950–2850 cm^−1^ region are attributed to asymmetric and symmetric stretching vibrations of aliphatic C–H bonds in –CH_2_– and –CH_3_ groups. The bands at approximately 1450–1465 and 1375–1385 cm^−1^ correspond to deformation vibrations of methylene and methyl groups, respectively. A weak feature near 1600 cm^−1^ may be associated with aromatic structures in the bitumen matrix [11,12,13].

The oil refinery sludge exhibits similar hydrocarbon-related bands, consistent with the presence of an organic hydrocarbon-containing fraction. Additional features in the 800–1200 cm^−1^ region may be associated with oxygen-containing groups and inorganic constituents of the sludge. The SBS spectrum contains bands characteristic of styrene and butadiene units, particularly in the approximately 699–760 and 910–970 cm^−1^ regions. In the modified binder, these features overlap with the absorption bands of bitumen and oil sludge; therefore, the contribution of SBS cannot be evaluated from isolated peaks alone.

Titanium slag and cyclone dust exhibit their main absorption features at lower wavenumbers, where vibrations associated with metal–oxygen and oxide–silicate structures may occur. Their spectral contributions overlap within the approximately 500–1100 cm^−1^ region. Consequently, the FTIR spectra are consistent with the presence of inorganic components; however, FTIR does not provide a complete determination of their phase or elemental composition. In the present study, XRD and XRF were applied to the prepared binders rather than to the individual industrial by-products.

As shown in Figure 3b, the 75:25 binder retains the principal hydrocarbon absorption bands observed in the control bitumen. This indicates that the organic bitumen phase remains the principal contributor to the FTIR spectrum after modification. Differences in band shape and relative intensity, particularly in the approximately 900–1100 and 1450–1600 cm^−1^ regions, may reflect overlapping contributions from SBS, oil sludge, and mineral components, as well as changes in their local physicochemical environment. However, FTIR alone does not allow the spatial distribution or phase continuity of these components to be determined.

No distinct new absorption bands were observed in the modified binder. In particular, no pronounced increase was detected in the carbonyl region at approximately 1700–1730 cm^−1^. Within the limitations of the qualitative FTIR comparison, this suggests that the preparation procedure did not produce pronounced oxidation-related changes detectable by this method. Overall, the preservation of the main bitumen bands and the absence of clearly identifiable new functional groups are consistent with a modification process governed predominantly by physical blending, polymer swelling, and possible weak physicochemical associations rather than by the formation of new covalent structures. Direct confirmation of polymer dispersion, particle distribution, and phase morphology would require complementary microscopic and rheological investigations.

### 3.2. TGA/DSC Analysis

**Figure 4:**
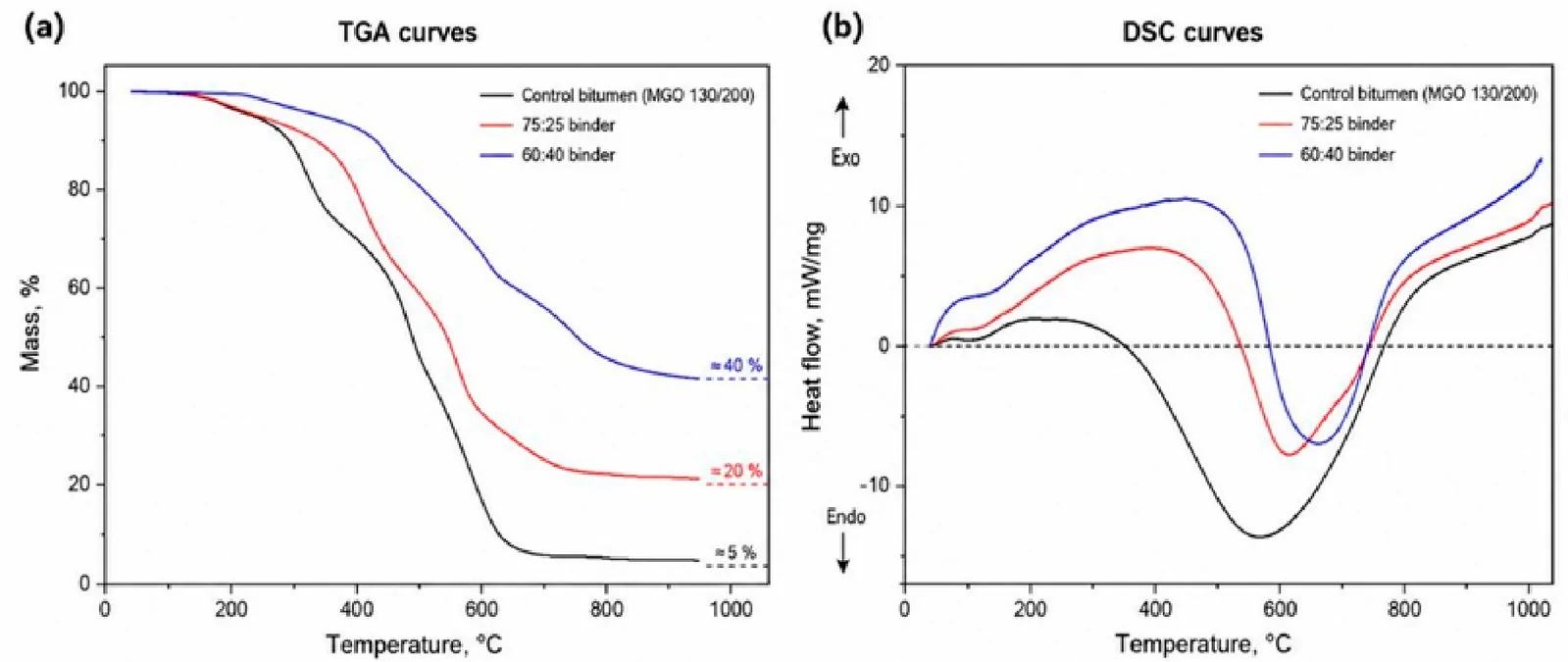
TGA/DSC curves of selected bitumen binders: (**a**) TGA curves of control bitumen, 75:25, and 60:40 binders; (**b**) DSC curves of control bitumen, 75:25, and 60:40 binders. The dashed horizontal line in (**b**) indicates the zero heat-flow reference (0 mW/mg).

The thermal behavior of the control bitumen, the selected 75:25 binder, and the 60:40 binder with the highest modifying-system content was evaluated by TGA/DSC analysis, as shown in Figure 4. The control sample was used as the reference organic binder, the 75:25 composition represented the formulation selected on the basis of the combined test results, and the 60:40 composition was included to evaluate the thermal response associated with the highest modifying-system content.

As shown in Figure 4a, the control bitumen exhibits thermal behavior typical of an organic hydrocarbon binder, with the main mass loss associated with the decomposition and volatilization of the bituminous organic phase. This behavior is consistent with the complex colloidal nature of bitumen, in which thermal degradation involves the progressive decomposition of lighter fractions, resins, and asphaltene-rich components [18].

The 75:25 binder shows a different mass-loss profile compared with the control sample. The incorporation of SBS, oil sludge, and mineral by-products changes the temperature-dependent mass-loss behavior and increases the amount of residue remaining after heating. The final residue of the 75:25 binder was approximately 20%, compared with approximately 5% for the control bitumen. This difference is consistent with the presence of inorganic components and thermally stable carbonaceous products in the modified binder. However, the residue value alone does not provide information about phase continuity, component dispersion, or deformation ability.

The 60:40 binder exhibited the highest final residue, approximately 40%, which is consistent with its higher content of mineral and organo-mineral modifying components. Nevertheless, a higher residual mass should not be interpreted as direct evidence of improved thermal or mechanical performance. In multicomponent bitumen binders, the residue may contain inorganic particles together with carbonaceous products formed during thermal decomposition. Therefore, the TGA results should be interpreted jointly with XRD, XRF, and physical–mechanical data.

The DSC curves in Figure 4b show that the control and modified binders exhibit broad thermal events whose position, intensity, and shape vary with composition. These differences indicate that the introduction of the modifying system affects the overall thermal response of the binder. However, the individual thermal events cannot be assigned unambiguously to volatilization, polymer transformation, organic-phase decomposition, or mineral-related processes on the basis of the presented DSC curves alone. Such assignments would require complementary analysis of the individual components, derivative thermogravimetric curves, and more detailed determination of characteristic temperatures and enthalpy changes.

The comparison between the 75:25 and 60:40 binders demonstrates that increasing the modifying-system content produces a substantial increase in residual mass but does not by itself establish a more favorable binder organization or performance. The higher residue of the 60:40 composition reflects its greater thermally stable fraction, whereas its unfavorable deformation behavior is established by the penetration and elongation results discussed below. Consequently, the selection of the 75:25 composition cannot be based on TGA/DSC data alone and results from the combined evaluation of thermal, structural, adhesion, and physical–mechanical characteristics.

Overall, TGA/DSC analysis demonstrates composition-dependent changes in mass-loss behavior, residual mass, and thermal response after the incorporation of SBS, oil sludge, titanium slag, and cyclone dust. Similar composition-dependent changes in the thermal degradation behavior of modified and rejuvenated bitumen binders have been reported in previous studies [19,20]. The 75:25 binder showed an intermediate residual fraction, whereas the 60:40 binder showed the highest residue associated with the greatest modifying-system content. These findings do not demonstrate synergy or determine an optimum composition independently; rather, they provide thermal evidence that must be considered together with the other physicochemical and performance results. Among the formulations investigated, the 75:25 binder was subsequently identified as the most balanced composition on the basis of the complete experimental dataset.

### 3.3. XRD Analysis

**Figure 5:**
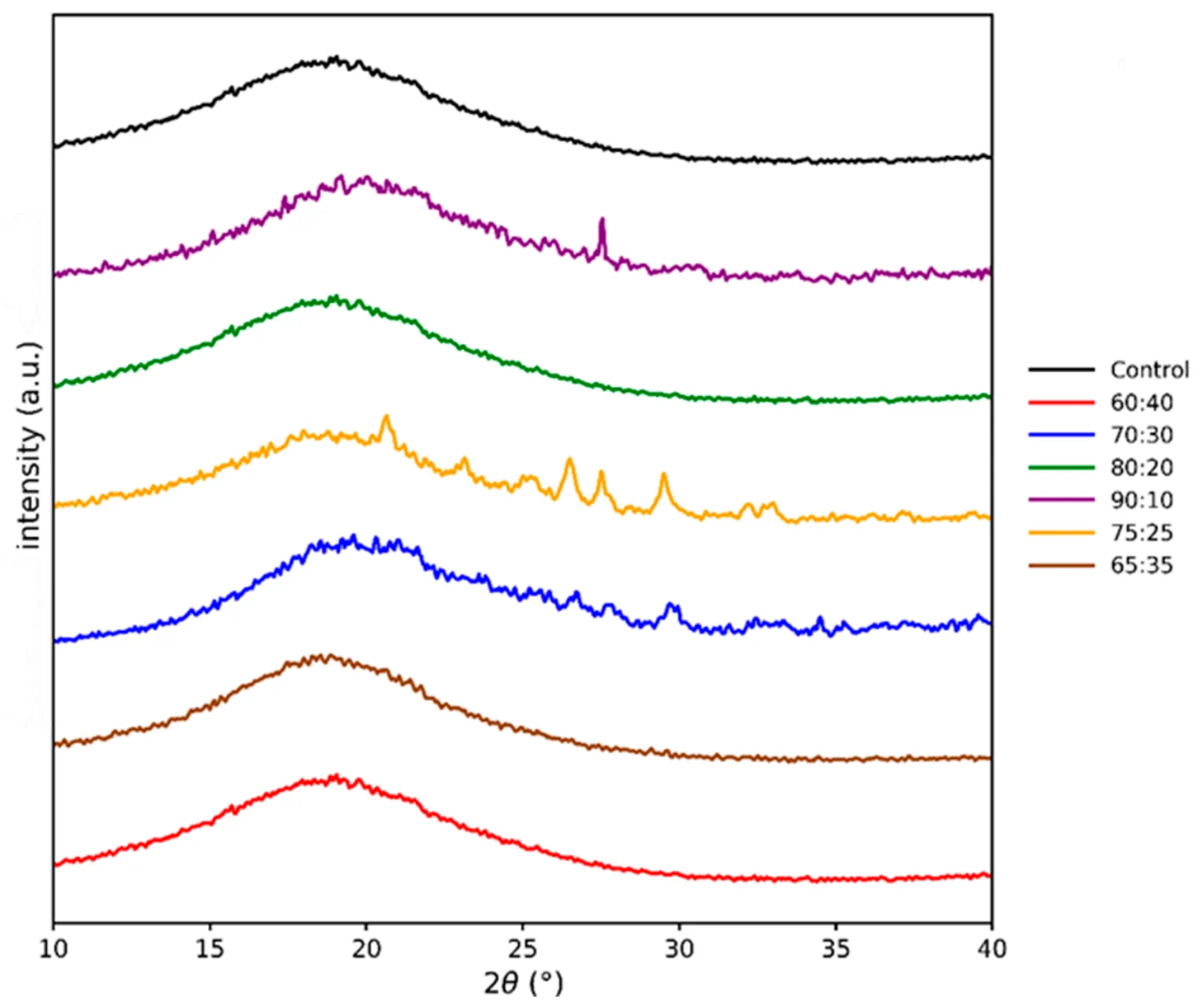
XRD patterns of the control bitumen and polymer–organic–mineral modified binders.

The XRD patterns of the control bitumen and all polymer–organic–mineral modified binders are shown in Figure 5. The patterns were compared to evaluate composition-dependent changes in the diffuse and crystalline contributions to the diffraction response.

The control bitumen shows a predominantly broad diffuse profile, which is consistent with the largely amorphous character of the organic bitumen phase. This behavior agrees with the colloidal and structurally heterogeneous nature of bituminous binders, in which long-range crystalline order is limited and the diffraction response is dominated by broad scattering from the organic matrix [21,22,23].

The 75:25 binder exhibits several local reflections superimposed on the broad diffuse background, mainly in the approximate regions of 21–22°, 23–24°, 25–28°, 30°, and 33–34° 2θ. These reflections are likely associated with crystalline mineral constituents introduced with the metallurgical by-products. Since phase-specific identification was outside the scope of this study, the observed reflections were interpreted only as a qualitative indication of a crystalline mineral contribution and were not assigned to individual compounds. The preservation of the broad diffuse background indicates that the amorphous organic contribution remains substantial, but it does not establish the continuity or spatial distribution of the bitumen phase.

The 60:40 binder, despite containing the highest amount of the modifying system, does not show an obvious proportional increase in the prominence of narrow reflections. Its pattern remains predominantly diffuse within the investigated 2θ range. This observation indicates that the observable diffraction features do not vary monotonically with the nominal modifying-system content. This behavior may reflect differences in the crystallinity of the introduced constituents and overlap between their reflections and the broad organic background. Therefore, the pattern of the 60:40 binder cannot be used alone to determine mineral-particle dispersion, agglomeration, or interaction with the organic–polymer phase.

The comparison among the investigated compositions shows a non-monotonic variation in the observable crystalline reflections. Within the qualitative comparison, the 75:25 binder displays the most distinct local reflections, whereas the 60:40 composition remains largely diffuse despite its higher modifier content. This result indicates that the diffraction response depends on more than the nominal amount of added mineral material. It does not demonstrate that the 75:25 binder possesses a more ordered, homogeneous, or intrinsically more effective structure.

Overall, the XRD results indicate a broad amorphous contribution together with detectable crystalline reflections in several modified binders. These observations complement the FTIR, TGA/DSC, and XRF results by supporting the presence of crystalline mineral constituents in the multicomponent binders. The XRD data alone do not establish phase continuity, particle distribution, or the superiority of a particular formulation. The selection of the 75:25 binder for the experimental asphalt concrete sections was therefore based on the combined physicochemical, adhesion, and physical–mechanical results rather than on its diffraction pattern alone.

### 3.4. XRF Analysis

X-ray fluorescence analysis was performed to evaluate the semi-quantitative surface elemental profile associated with the mineral components present in the modified bitumen binders. Particular attention was paid to Pb and Ti, which were considered characteristic elemental indicators of the lead-furnace cyclone dust and titanium slag, respectively. The selected elements detected in the investigated compositions are presented in Table 2.

Pb and Ti produced the highest relative XRF values among the selected reported elements in all modified binders. Their detection is consistent with the presence of Pb- and Ti-containing metallurgical components on the analyzed sample surfaces. For the 80:20–60:40 compositions, the relative Pb values varied within a comparatively narrow range of 39.62–41.55%, whereas the Ti values ranged from 32.64 to 34.72%. Within the limitations of the semi-quantitative surface measurement, these formulations therefore showed broadly similar relative Pb–Ti profiles.

The 90:10 composition showed a different relative surface profile, with a Pb value of 43.89% and a Ti value of 17.44%. Higher relative Fe and Zn values were also recorded for this sample. These differences may reflect local variation in the mineral particles present at the analyzed surface, particularly because the 90:10 binder contained the lowest total amount of the modifying system. However, portable XRF measurements of heterogeneous organic–mineral materials are sensitive to the analyzed location and matrix effects. Therefore, the observed differences should not be interpreted as quantitative evidence of bulk compositional variation or particle distribution throughout the binder.

The 75:25 binder showed relative Pb and Ti values of 40.67% and 33.28%, respectively. These results are qualitatively compatible with the crystalline mineral contribution observed in its XRD pattern. Nevertheless, the two methods provide different information: XRF indicates the elements detected at the analyzed surface, whereas XRD reflects the diffraction response of crystalline constituents. Neither method alone establishes the spatial distribution of the mineral particles or the continuity of the bitumen phase.

Overall, the XRF results support the presence of Pb-, Ti-, Fe-, Zn-, and other metal-containing constituents on the analyzed surfaces of the modified binders. For the 80:20–60:40 formulations, the selected relative elemental profiles were generally comparable, while the 90:10 sample showed a more distinct surface profile. The XRF data were used primarily as qualitative and semi-quantitative evidence of the introduced metallurgical components, rather than for determination of their absolute bulk concentrations, chemical state, or spatial distribution. Furthermore, XRF does not provide information on the mobility or leachability of the detected metals; these aspects require separate environmental assessment before conclusions regarding long-term field safety can be made.

### 3.5. Penetration, Softening Point, and Tensile Elongation

The consistency, softening behavior, and tensile deformation characteristics of the control bitumen and polymer–organic–mineral modified binders are presented in Table 3. All compositions were included to evaluate the composition-dependent response of the binders. These results were considered together with the adhesion and physicochemical data when selecting the most balanced formulation among those investigated.

The penetration depth decreased markedly for the 90:10–65:35 binders compared with the control bitumen. The control sample exhibited a penetration depth of 28.2 mm, whereas these modified compositions showed values ranging from 5.6 to 8.6 mm. Under the applied test conditions, lower penetration values indicate greater resistance to local needle penetration and, consequently, a firmer binder consistency. However, the observed response cannot be attributed independently to SBS, oil sludge, or the mineral components because these constituents were introduced as a combined modifying system. The lowest penetration depth was recorded for the 70:30 binder.

The softening point increased for all modified binders. The control bitumen had a softening point of 32.5 °C, whereas the modified compositions showed values between 44.0 and 104.0 °C. The highest value was obtained for the 80:20 binder, followed by the 90:10 and 70:30 compositions. A higher softening point indicates reduced susceptibility to softening under the conditions of the ring-and-ball test. However, this parameter should not be interpreted independently as direct evidence of rutting resistance, complete rheological performance, or brittleness.

The maximum elongation values also varied substantially among the investigated compositions. The control bitumen reached 500.02 mm, indicating the greatest ability to sustain extension before failure under the applied tensile test conditions. Among the modified binders, the 65:35 composition exhibited the highest elongation of 420.08 mm, followed by the 70:30 and 75:25 binders with values of 369.28 and 355.66 mm, respectively. The 90:10 and 80:20 binders showed considerably lower elongation, while the 60:40 composition exhibited the lowest value of 71.63 mm. These results characterize tensile deformation capacity under the applied test conditions and should not be regarded as a complete assessment of low-temperature cracking or fatigue resistance.

The 75:25 binder exhibited a penetration depth of 8.6 mm, a softening point of 65.0 °C, and a maximum elongation of 355.66 mm, corresponding to approximately 71% of the control value. This combination indicates that the binder retained substantial tensile deformability while showing a lower penetration depth and a higher softening point than the control bitumen.

Nevertheless, the results presented in Table 3 alone do not establish the 75:25 composition as uniquely superior. In particular, the 70:30 binder exhibited a higher softening point, slightly greater elongation, and the lowest penetration depth. Therefore, the selection of the 75:25 formulation was based on the complete experimental dataset, including adhesion, physicochemical characteristics, processing behavior, and the adopted balance between consistency, resistance to softening, deformation capacity, and modifier content.

The 60:40 binder showed a penetration depth of 37.2 mm, which was higher than that of both the control and the other modified compositions. This result was accompanied by a comparatively low softening point of 44.0 °C and the lowest elongation value of 71.63 mm. Therefore, the behavior of the 60:40 binder cannot be described simply as uniform softening: increased local needle penetration occurred simultaneously with a substantial loss of tensile deformation capacity.

A plausible explanation is an unfavorable balance among the components at the highest modifying-system content. In the 60:40 formulation, the proportion of base bitumen was reduced to 60 wt.%, while the contents of oil sludge and mineral by-products were the highest among the investigated binders. The hydrocarbon-containing fraction of the oil sludge may have exerted a local plasticizing effect, contributing to the increased penetration depth. At the same time, the high content of mineral particles may have restricted tensile deformation and increased compositional heterogeneity. In addition, the SBS content may have been insufficient relative to the total amount of organic and mineral by-products to provide uniform polymer reinforcement throughout the binder. These combined effects may explain the simultaneous increase in penetration and decrease in elongation. However, the internal phase morphology and polymer-network distribution were not directly assessed; therefore, this explanation should be regarded as a plausible interpretation rather than a confirmed structural mechanism.

Overall, the penetration, softening-point, and elongation results demonstrate a composition-dependent and non-monotonic response of the multicomponent binders. Increasing the modifying-system content did not produce a progressive improvement in all measured properties. The 60:40 composition showed the least favorable combined response, whereas the 75:25 binder was selected as the most balanced formulation among those investigated on the basis of the complete experimental dataset rather than on any single conventional property. Accordingly, the 75:25 composition should not be considered universally optimal for asphalt pavement applications, but rather the most balanced formulation under the preparation conditions and selection criteria adopted in this study.

The observed trends are generally consistent with previous studies showing that SBS modification can increase resistance to softening and permanent deformation [4,5,6], while petroleum-derived sludge modifiers may alter binder consistency and deformability [7,8]. However, direct numerical comparison with conventional SBS-modified binders is limited because the present study used adapted penetration and tensile-deformation procedures. In addition, an SBS-only reference binder was not included. Therefore, the significance of the 75:25 formulation should be considered in terms of the balance of properties within the investigated multicomponent series rather than as evidence of superiority over conventional SBS-modified bitumen.

### 3.6. Qualitative Adhesion Assessment

Adhesion was evaluated qualitatively based on the retention of the bitumen film on the surface of crushed stone after water–thermal exposure. The assessment was intended to compare the resistance of the binder films to visible peeling under the applied test conditions. The results are summarized in Table 4.

The qualitative adhesion assessment showed high retention of the bitumen film for the 90:10, 80:20, and 75:25 compositions. Because these three binders were assigned to the same qualitative category, the applied test does not provide a basis for ranking their adhesion performance within this group.

The tested specimens of the 70:30 binder showed different degrees of film retention and were therefore classified as variable. The 65:35 composition exhibited pronounced destruction and peeling of the bitumen film and was classified as having low adhesion. The 60:40 binder retained the film with minor local defects and was therefore classified as satisfactory.

Among the formulations that exhibited high qualitative film retention, the 75:25 binder also retained substantial tensile deformation capacity and showed a higher softening point and lower penetration depth than the control bitumen. These adhesion results therefore contributed to, but did not independently determine, the selection of the 75:25 composition as the most balanced formulation among those investigated.

### 3.7. Rationale for Selecting the 75:25 Binder

The preferred formulation was selected using a multi-criteria approach because no individual test provides a complete assessment of a multicomponent bitumen binder. The selection was therefore based on the combined evaluation of penetration depth, softening point, tensile elongation, qualitative adhesion, and physicochemical characterization.

FTIR analysis showed that the 75:25 binder retained the principal hydrocarbon absorption bands of the control bitumen and exhibited no clear increase in the carbonyl region. Within the limitations of the qualitative spectral comparison, these results indicate that no pronounced oxidation-related changes detectable by FTIR occurred during preparation. The FTIR data were therefore used to characterize the general chemical environment of the binder rather than to determine component distribution or phase morphology.

The TGA/DSC results showed that the introduction of the modifying system altered the mass-loss behavior, thermal response, and amount of residue remaining after heating. The higher residue of the 60:40 binder demonstrated that an increased thermally stable fraction did not correspond to a more favorable combination of conventional binder properties. Consequently, the thermal results were considered together with the penetration, softening-point, and elongation data rather than being used as an independent selection criterion.

The XRD and XRF results provided qualitative evidence of crystalline mineral constituents and Pb-, Ti-, Fe-, and Zn-containing components in the modified binders. The 75:25 composition exhibited detectable crystalline reflections and a relative Pb–Ti surface profile consistent with the presence of the introduced metallurgical components. However, these methods did not independently establish particle distribution, phase continuity, or superiority of the 75:25 formulation.

The conventional-property results showed that the 75:25 binder had a penetration depth of 8.6 mm, a softening point of 65.0 °C, and a maximum elongation of 355.66 mm. Thus, compared with the control bitumen, it combined lower needle penetration and a higher softening point with retention of approximately 71% of the original tensile elongation. The qualitative adhesion test also classified the 75:25 binder as having high film retention after water–thermal exposure.

The results of the individual tests did not identify the 75:25 formulation as uniquely superior. For example, the 70:30 binder exhibited a higher softening point, slightly greater elongation, and lower penetration depth. However, its qualitative adhesion response was variable, whereas the 75:25 binder combined high film retention with substantial elongation and an intermediate modifying-system content. The 90:10 and 80:20 binders also showed high qualitative adhesion, but their considerably lower elongation values indicated a greater loss of tensile deformation capacity. The 65:35 binder retained high elongation but showed low qualitative adhesion, while the 60:40 composition exhibited the least favorable combination of high penetration and low elongation.

Accordingly, the 75:25 binder was selected as the most balanced formulation among the compositions investigated under the adopted preparation and testing conditions. This selection reflects the combined balance of consistency, resistance to softening, tensile deformation capacity, qualitative adhesion, and modifier content rather than the maximum value of any individual parameter. The 75:25 composition was therefore used for the preparation of the experimental asphalt concrete sections.

### 3.8. Preliminary Small-Scale Asphalt Concrete Trial

The preliminary technological feasibility of using the selected 75:25 binder in asphalt concrete was evaluated through the preparation of small-scale experimental sections. The purpose of this stage was to examine the behavior of the modified binder during mixture preparation, aggregate coating, placement, compaction, and initial surface formation under the adopted experimental conditions rather than to assess long-term pavement performance.

Two comparative sections were prepared: a control section containing the base bitumen and an experimental section containing the 75:25 polymer–organic–mineral binder. Both sections had approximately the same dimensions and were constructed on a compacted crushed stone–sand base using the same general preparation and placement procedure. A coarse-grained asphalt concrete layer with a thickness of approximately 6–7 cm was placed over the prepared base.

During mixture preparation, the asphalt concrete containing the 75:25 binder could be mixed, used to coat the mineral aggregate, placed, and compacted without immediate handling difficulties. No obvious binder separation or visible local accumulation of the mineral-modifying components was observed during placement. These observations indicate acceptable short-term processing behavior under the applied conditions but do not establish storage stability, component compatibility, or uniform particle distribution within the mixture.

After compaction, the experimental section formed a generally uniform visible surface. No obvious binder bleeding, pronounced local soft zones, or visible aggregate stripping were observed during the initial inspection. Both the control and modified mixtures could be placed and compacted using the adopted procedure. However, density, air-void content, and degree of compaction were not determined quantitatively.

Surface macrotexture was assessed using an adapted sand patch procedure based on ST RK 1279-2013 [17]. Before testing, the surface was cleaned with a brush to remove dust, loose sand, and poorly attached particles. A fixed volume of 25 cm^3^ of dry sand was placed on the prepared surface and distributed using a flat circular tool until the surface depressions were filled and an approximately circular patch was formed. The diameter of the resulting sand patch was measured in four intersecting directions, and the mean diameter was calculated as:

$$D_{\mathrm{mean}}=\frac{D_{1}+D_{2}+D_{3}+D_{4}}{4}$$

The mean texture depth was calculated using:

$$h=\frac{40V}{\pi D_{mean}^{2}}$$

where *h* is the mean texture depth in millimetres, *V* is the sand volume in cubic centimetres, and *D*_mean_ is the mean sand patch diameter in centimetres.

**Figure 6:**
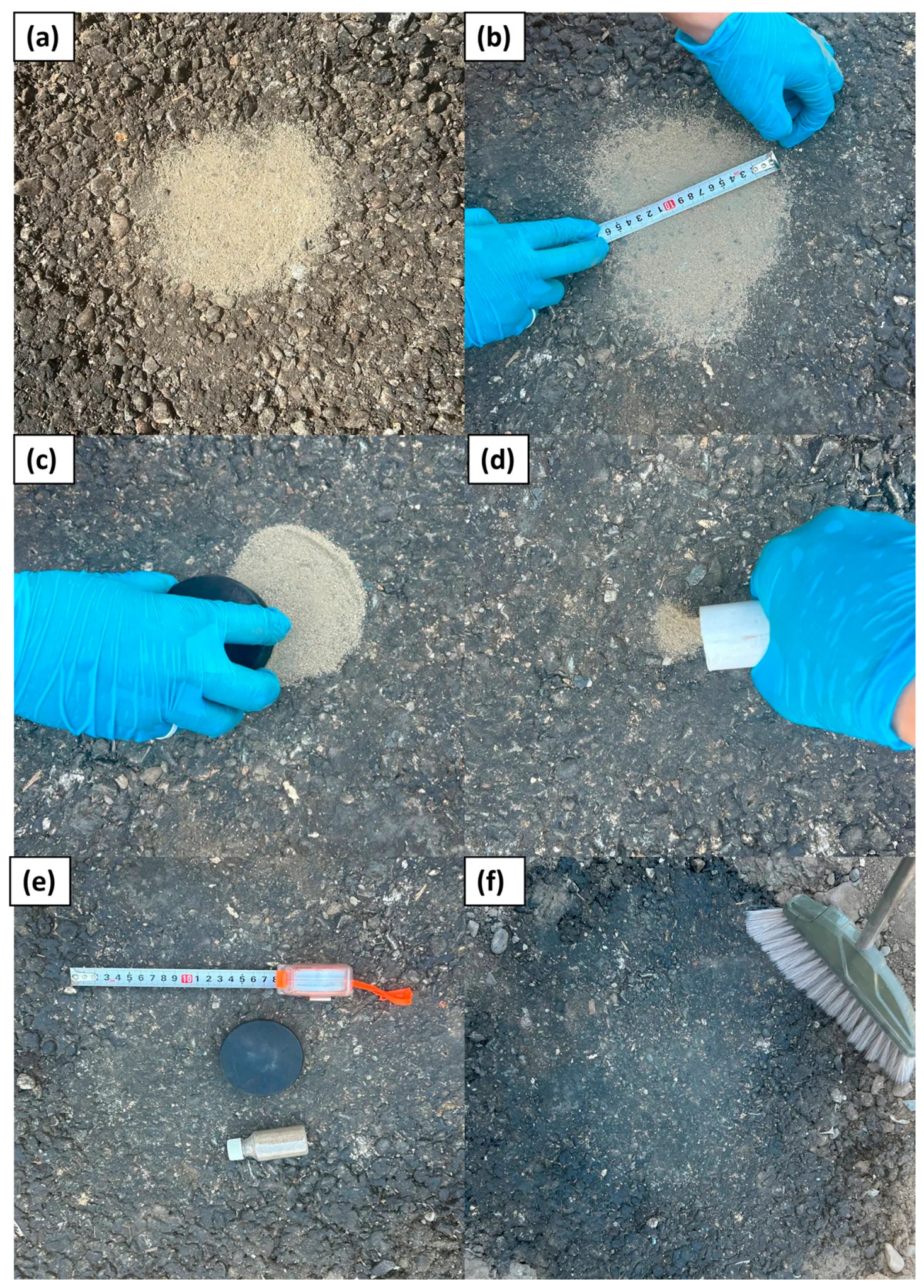
Sand patch procedure used for the preliminary assessment of the experimental asphalt concrete surface: (**a**) placement of the sand material; (**b**) measurement of the sand patch diameter; (**c**) spreading of the sand using a flat circular tool; (**d**) dosing of the sand; (**e**) tools used during the procedure; and (**f**) cleaning of the surface before testing.

The sand patch procedure is illustrated in Figure 6, and the corresponding results are summarized in Table 5.

The control section exhibited a smaller mean sand patch diameter and a greater calculated texture depth than the section containing the 75:25 binder. Under the applied measurement conditions, the control surface therefore showed a more pronounced local macrotexture, whereas the modified section exhibited a somewhat smoother surface. Nevertheless, both sections showed measurable aggregate-related surface texture.

The sand patch results should be interpreted as a preliminary local comparison. The four diameter readings for each section represented directional measurements of the same sand patch rather than independent replicate tests. In addition, the measurements were performed on small experimental sections without application of the complete sampling scheme prescribed for full-scale road-surface evaluation. Therefore, the observed difference cannot be regarded as statistically established or used to demonstrate formal compliance with pavement macrotexture requirements.

The friction coefficient was not measured in the present study. Consequently, the obtained texture-depth values should not be interpreted as direct evidence of skid resistance, drainage performance, or compliance with the friction-related threshold values given in ST RK 1279-2013.

Overall, the small-scale trial showed that the 75:25 binder could be incorporated into asphalt concrete and processed under the adopted experimental conditions without obvious immediate technological defects. The sand patch results provide preliminary quantitative information on local surface macrotexture. Further studies should include replicated macrotexture measurements, density and air-void determination, rutting resistance, water sensitivity, fatigue behavior, aging resistance, environmental leaching, and long-term monitoring under traffic and climatic exposure.

## 4. Conclusions

A multicomponent polymer–organic–mineral bitumen binder based on MGO 130/200 bitumen, SBS, oil refinery sludge, titanium slag, and lead-furnace cyclone dust was investigated over a range of modifying-system contents. The results demonstrated a non-monotonic composition-dependent response, indicating that increasing the modifier content did not progressively improve all binder properties.

Among the investigated formulations, the 75:25 binder provided the most balanced combination of conventional properties, with a penetration depth of 8.6 mm, a softening point of 65.0 °C, and a maximum elongation of 355.66 mm, corresponding to approximately 71% of the control value. It also showed high qualitative film retention after water–thermal exposure. FTIR, TGA/DSC, XRD, and XRF analyses confirmed composition-dependent physicochemical changes and the presence of introduced mineral constituents, but did not establish specific molecular interactions, phase continuity, or true synergistic effects.

The preliminary small-scale asphalt concrete trial confirmed that the 75:25 binder could be prepared, placed, and compacted under the adopted experimental conditions without obvious immediate technological defects. However, the study did not include single-modifier or binary reference binders, DSR or BBR measurements, or long-term field evaluation. These limitations should be considered when interpreting the present results and assessing the broader applicability of the proposed binder.

## Figures and Tables

**Table 1:** Composition of the control and polymer–organic–mineral modified bitumen binders.

Sample	Bitumen, wt.%	Total Modifying System, wt.%	SBS, wt.%	Oil Sludge, wt.%	Titanium Slag, wt.%	Cyclone Dust, wt.%	Added Metallurgical Mineral Fraction, wt.%
Control	100	0	0	0	0	0	0
90:10	90	10	3	2	3	2	5
80:20	80	20	4	5	6	5	11
75:25	75	25	4.5	6.5	8	6	14
70:30	70	30	5	8	10	7	17
65:35	65	35	5	10	11.5	8.5	20
60:40	60	40	5	12	13	10	23

Note: The total modifying-system content is the sum of SBS, oil sludge, titanium slag, and cyclone dust. The added metallurgical mineral fraction is the combined content of titanium slag and lead-furnace cyclone dust and does not include the oil sludge.

**Table 2:** Semi-quantitative relative elemental values obtained for the polymer–organic–mineral modified bitumen binders by portable XRF analysis.

Sample	Pb, %	Ti, %	Fe, %	Cd, %	Zn, %	As, %	Cr, %	Zr, %
Control	n.d.	n.d.	n.d.	n.d.	n.d.	n.d.	n.d.	n.d.
90:10	43.89	17.44	15.34	1.96	8.40	1.69	1.71	0.98
80:20	41.55	32.64	8.80	5.79	3.94	1.90	1.24	0.77
75:25	40.67	33.28	10.21	5.83	3.94	0.83	1.20	0.77
70:30	40.39	34.47	9.67	5.14	3.82	1.10	1.28	0.71
65:35	39.62	34.72	8.63	5.73	3.73	1.75	1.49	0.82
60:40	40.52	34.10	9.84	5.25	3.86	1.05	1.31	0.74

n.d.—not detected under the applied measurement conditions. Only selected detected elements are presented. The values are instrument-reported semi-quantitative relative surface values and should not be interpreted as bulk mass fractions of the complete binder, because the organic matrix and unreported elements are not represented in the table.

**Table 3:** Penetration depth, softening point, and maximum tensile elongation of the control and modified bitumen binders.

Sample	Penetration Depth, mm	Softening Point, °C	Maximum Elongation, mm	Observed Combination of Properties
Control	28.2	32.5	500.02	High elongation and low softening point
90:10	6.1	92.5	124.11	Low penetration, high softening point, and reduced elongation
80:20	7.2	104.0	155.39	Highest softening point and relatively low elongation
75:25	8.6	65.0	355.66	Intermediate softening behavior with preserved elongation
70:30	5.6	76.5	369.28	Lowest penetration with relatively high elongation
65:35	7.1	69.5	420.08	Highest elongation among the modified binders
60:40	37.2	44.0	71.63	Highest penetration and lowest elongation among the tested binders

**Table 4:** Qualitative adhesion assessment of the polymer–organic–mineral modified bitumen binders.

Sample	Adhesion Assessment
Control	n.d.
90:10	High
80:20	High
75:25	High
70:30	Variable
65:35	Low
60:40	Satisfactory

n.d.—not determined. Adhesion categories: High—bitumen film retained without visible peeling; Variable—different degrees of film retention observed in the tested specimens; Low—pronounced destruction and peeling of the film; Satisfactory—film retained with minor local defects.

**Table 5:** Preliminary sand patch results for the control and modified asphalt concrete sections.

Asphalt Concrete Section	Mean Patch Diameter, cm	Mean Texture Depth, mm
Section containing the 75:25 modified binder	11.10	2.58
Control section containing the base bitumen	10.03	3.17

## Data Availability

The original contributions presented in this study are included in the article. Further inquiries can be directed to the corresponding author.
